# ABC Transporter Genes Show Upregulated Expression in Drug-Resistant Clinical Isolates of *Candida auris*: A Genome-Wide Characterization of ATP-Binding Cassette (ABC) Transporter Genes

**DOI:** 10.3389/fmicb.2019.01445

**Published:** 2019-07-16

**Authors:** Mohd Wasi, Nitesh Kumar Khandelwal, Alexander J. Moorhouse, Remya Nair, Poonam Vishwakarma, Gustavo Bravo Ruiz, Zoe K. Ross, Alexander Lorenz, Shivaprakash M. Rudramurthy, Arunaloke Chakrabarti, Andrew M. Lynn, Alok K. Mondal, Neil A. R. Gow, Rajendra Prasad

**Affiliations:** ^1^School of Life Sciences, Jawaharlal Nehru University, New Delhi, India; ^2^MRC Centre for Medical Mycology, University of Aberdeen, Aberdeen, United Kingdom; ^3^Amity Institute of Biotechnology and Integrative Sciences and Health, Amity University Gurugram, Gurgaon, India; ^4^School of Computational and Integrative Science, Jawaharlal Nehru University, New Delhi, India; ^5^The Institute of Medical Sciences, School of Medicine, Medical Sciences and Nutrition, University of Aberdeen, Aberdeen, United Kingdom; ^6^Department of Medical Microbiology, Post Graduate Institute of Medical Education and Research, Chandigarh, India; ^7^School of Biosciences, University of Exeter, Exeter, United Kingdom

**Keywords:** C*andida auris*, multidrug resistance, ABC proteins, drug efflux pumps, fluconazole

## Abstract

ATP-binding cassette (ABC) superfamily members have a key role as nutrient importers and exporters in bacteria. However, their role as drug exporters in eukaryotes brought this superfamily member to even greater prominence. The capacity of ABC transporters to efflux a broad spectrum of xenobiotics represents one of the major mechanisms of clinical multidrug resistance in pathogenic fungi including *Candida* species. *Candida auris*, a newly emerged multidrug-resistant fungal pathogen of humans, has been responsible for multiple outbreaks of drug-resistant infections in hospitals around the globe. Our study has analyzed the entire complement of ABC superfamily transporters to assess whether these play a major role in drug resistance mechanisms of *C. auris*. Our bioinformatics analyses identified 28 putative ABC proteins encoded in the genome of the *C. auris* type-strain CBS 10913T; 20 of which contain transmembrane domains (TMDs). Quantitative real-time PCR confirmed the expression of all 20 TMD transporters, underlining their potential in contributing to the *C. auris* drug-resistant phenotype. Changes in transcript levels after short-term exposure of drugs and in drug-resistant *C. auris* isolates suggested their importance in the drug resistance phenotype of this pathogen. *CAUR_02725* orthologous to *CDR1*, a major multidrug exporter in other yeasts, showed consistently higher expression in multidrug-resistant strains of *C. auris*. Homologs of other ABC transporter genes, such as *CDR4*, *CDR6*, and *SNQ2*, also displayed raised expression in a sub-set of clinical isolates. Together, our analysis supports the involvement of these transporters in multidrug resistance in *C. auris*.

## Introduction

The ATP-binding cassette (ABC) proteins are one of the largest superfamily of proteins found in both prokaryotes and higher eukaryotes. The eukaryotic ABC transporters are promiscuous exporters and can extrude a variety of substrates including metals, drugs, xenobiotics, lipids, and other cellular metabolites (Prasad and Goffeau, 2012). A large number of ABC proteins are multidrug transporters and function as efflux pumps. Rapid active drug extrusion from cells represents one of the major mechanisms of multidrug resistance, frequently encountered in organisms ranging from bacteria to mammals (Prasad and Goffeau, 2012). However, the function of ABC proteins is not limited to detoxification or drug expulsion, since many of its members perform diverse cellular functions. Among others, these include roles in vacuole fusion, maintaining mitochondrial integrity, lipid translocation, and pheromone secretion (Khandelwal et al., 2016; Prasad et al., 2016). Notably, the ABC proteins play major roles in various human diseases, such as cystic fibrosis, Tangier’s disease, adrenoleukodystrophy, and cancer (Dean et al., 2001b).

*Candida* species are the fourth most common cause of fungal blood stream infections, and among them *Candida albicans* is the most prevalent agent of superficial and systemic disease (Wisplinghoff et al., 2004; Tortorano et al., 2006). However, recent global emergence of multidrug-resistant *Candida auris* has become a major health concern (Cortegiani et al., 2018). Compared to other pathogenic *Candida* species, *C. auris* has unique features, which make it difficult to treat and eradicate from intensive care hospital wards (Forsberg et al., 2019). This fungus is mostly associated with clonal outbreaks that spread rapidly through health care facilities. *C. auris* is frequently resistant to commonly used frontline antifungals of different classes. An initial study on *C. auris* showed that it is phylogenetically related to *Candida haemulonii* and has the largest numbers of orthologs with *Candida lusitaniae* (Chatterjee et al., 2015).

Whole-genome sequence (WGS) analyses of *C. auris* isolates from different countries led to a subdivision into four distinct clades specific to a geographic area, thus indicating the simultaneous emergence of *C. auris* in different continents (Lockhart et al., 2017). Based on the geographically restricted area of isolation, the authors have classified the clades as follows: clade I comprises isolates from India and Pakistan, clade II from Japan and South Korea, clade III from South Africa, and clade IV from Venezuela (Schelenz et al., 2016; Lockhart et al., 2017; Muñoz et al., 2018; Rhodes et al., 2018). As per this classification, the *C. auris* strain, CBS 10913T belongs to clade II (Japan and South Korea) and the three Indian clinical-resistant isolates used in this study probably belong to clade I (India and Pakistan). The different clades are differentiated by thousands of single-nucleotide polymorphisms (SNPs), whereas genetic differences within each geographic clade are minimal (Lockhart et al., 2017). Importantly, not much is known about phenotypic differences between isolates from different *C. auris* clades. Initial WGS of a *C. auris* isolate has revealed that approximately 2.4% of its genes encode ABC and major facilitator superfamily (MFS) transporters along with other transporters like oligopeptide transporters and iron transporters, etc. (Chatterjee et al., 2015). Two recent simultaneously published reports confirmed the role of ABC transporter *CDR1* and MFS transporter *MDR1* in azole resistance (Kim et al., 2019; Rybak et al., 2019).

Our study presents the identification and expression analysis of ABC proteins on a genome-wide scale in the pathogenic yeast *C. auris*. For this, we performed WGS of the *C. auris* type-strain, CBS 10913T (clade II – Japan and South Korea), recovered in 2009 from a Japanese patient with an ear infection (Satoh et al., 2009). Our bioinformatics analysis of the genome of CBS 10913T identified 28 putative ABC proteins. Based on phylogenetic analysis, domain organization, and following the nomenclature adopted by the Human Genome Organization (HUGO) scheme (Vasiliou et al., 2009), these proteins are classified into six subfamilies ABCB/MDR, ABCC/multidrug resistance-associated protein (MRP), ABCD/ALDp, ABCE/RLI, ABCF/YEF3, and ABCG/PDR. Among these, 20 contained transmembrane domains (TMDs). The TMD ABC transporters predominantly belong to the ABCG/PDR, ABCB/MDR, ABCC/MRP, and ABCD/ALDp subfamilies. Comparative phylogenetic analysis of the ABC proteins with *C. albicans*, *Saccharomyces cerevisiae*, and *Debaryomyces hansenii* revealed orthologous relationships and highlighted their conservation. We also demonstrate that the expression of various ABC transporters changes substantially in the presence of different antifungal drugs suggesting a potential role in drug resistance in *C. auris*. The comparative expression landscape of all transcripts of ABC transporters in the clade I multidrug-resistant hospital isolates of *C. auris* suggests a possible role of selected ABC proteins in conferring drug resistance.

## Materials and Methods

### Yeast Strains, Culture Conditions, and Antifungal Drugs

The *C. auris* type strain CBS 10913T was obtained from the CBS-KNAW fungal culture collection of the Westerdijk Fungal Biodiversity Institute, Utrecht, Netherlands. Although B11220 and CBS 10913T have apparently been isolated from the same original material they seem not to be completely identical. Additionally, their karyotypes are overall quite similar, but show some differences (Bravo Ruiz et al., 2019).

Three drug-resistant clinical isolates (*Isolate 1-NCCPF470150*, *Isolate 2-NCCPF 470156*, and *Isolate 3-NCCPF470114*) of *C. auris* were provided by the National Culture Collection of Fungal Pathogens, Postgraduate Institute of Medical Education & Research, Chandigarh, India. All yeast strains used in this study are listed in Supplementary Table S1. Yeasts were grown on yeast extract/peptone/dextrose (YEPD; Difco, Sparks, MD, United States). The antifungals amphotericin B (AMPB), terbinafine (TRB), and fluconazole (FLU) were procured from the Sigma Chemical Co. (St. Louis, MO, United States).

### Whole-Genome Sequencing (WGS) and Assembly

Genomic DNA of strain CBS 10913T was extracted from an overnight culture in YEPD broth using an established protocol. Briefly, cells were resuspended in equal volumes of extraction buffer (2% Triton X-100, 1% SDS, 100 mM NaCl, 10 mM Tris pH 8.0, 1mM EDTA) and phenol:chloroform:isoamyl alcohol (25:24:1). Vigorous shaking in the presence of acid-washed glass beads was applied to break the cell wall. After addition of a volume of 1× Tris–EDTA (TE pH 7.5) and centrifugation (5 min, 14,000 × *g*), the aqueous layer was mixed with 2.5 volumes of 100% ethanol to precipitate the DNA. Following centrifugation (2 min, 14,000 × *g*) the pelleted genomic DNA was suspended in 1 volume of 1× TE containing 250 μg/ml RNase A and incubated for 20 min at 37°C. DNA was then precipitated by adding 1/20 of a volume of 3 M sodium acetate and 2.5 volumes of 100% ethanol followed by centrifugation (10 min, 14,000 × *g*), finally DNA was resuspended in sterile water. Concentration and purity of the DNA preparation were determined on a NanoDrop^TM^ Spectrophotometer (Thermo Fisher Scientific, Waltham, MA, United States). A genomic library was prepared and barcoded at the Centre of Genome-Enabled Biology and Medicine (CGEBM, University of Aberdeen) using the Nextera DNA Library Preparation Kit (Illumina, Inc., San Diego, CA, United States), and WGS was performed on an Illumina MiSeq sequencer at CGEBM. Sequence reads were subjected to quality control inspection and trimming using FASTX-toolkit^1^ and FastQC^2^, and adaptor sequences were removed using Cutadapt (Martin, 2011). Velvet (Zerbino, 2010) and ABySS (Simpson et al., 2009) assemblers were used in standard and optimized mode with three sets of paired end reads: untrimmed reads, reads trimmed to a base quality score of 36, and to a base quality score of 28. CEGMA was used to conduct assembly quality control checks for genome completeness (Parra et al., 2007). The initial assembly was scaffolded using read alignments with BESST (Sahlin et al., 2014) and extended using IMAGE in PAGIT. The contiguity of the assembly was then improved by additional scaffolding against the published genome of the closely related species *C. lusitaniae* using Newbler aligner. Resulting contigs were curated to retain only contigs with read coverage >100 and <10,000 and a length of >1,000 bp, gap closing was performed using SSPACE-LongRead (Boetzer and Pirovano, 2014). The raw sequence files are available at https://www.ebi.ac.uk/ena/data/view/PRJEB29190 (ENA Accession No. PRJEB29190).

### Identification of ABC Proteins

A total of 5,279 proteins were identified in the genome of *C. auris* CBS 10913T. The HMM profile of the ABC-tran model (accession PF00005) obtained from the Pfam database^3^ was used as query against the proteins of *C. auris* in the HMM search program of HMMER package^4^ with default parameters (Finn et al., 2016). For further filtering of hits obtained after HMM search cut-off was decided on the basis of plots between domain scores and *E*-values. Domain (bit) scores above 69.4 and *E*-values below 1.3e−19 were considered positive for having an NBD domain. Sequences thus selected were taken forward as putative ABC proteins for detailed analysis.

### Retrieving of Sequences and Phylogenetic Analysis

For combined phylogenetic analysis *S. cerevisiae*, *C. albicans*, and *D. hansenii* sequences were retrieved from UniProt using previously published information (Wasi et al., 2018), and aligned with *C. auris* sequences by ClustalΩ using default parameters. The phylogenetic tree was constructed by MEGA 6.06 (Tamura et al., 2013) using the neighbor-joining method (Wasi et al., 2018) for the clustering, and the Poisson substitution for calculating the evolutionary distance. 1,000 bootstrap replicates were utilized to establish the tree topology. To calculate the sequence identities of ABC proteins among different species the web-based BLASTp program from NCBI^5^ was used.

### Subcellular Localization Prediction

To predict the subcellular localization of ABC transporters, we used WoLF PSORT^6^ and DEEPLOC^7^ software.

### RNA Isolation, cDNA Synthesis, and Quantitative Real-Time PCR

For the total RNA isolation, a primary culture was grown overnight to saturation. From this a secondary culture was inoculated in YEPD broth at an OD_600_ of 0.2, and grown for 6 h. The cultures were incubated for another 60 min with the indicated concentrations of drugs (Supplementary Table S2), and then collected by centrifugation, and washed with DEPC-treated water. Total RNA was isolated using an RNeasy Mini Kit (Qiagen, Hilden, Germany), following the manufacturer’s specifications. cDNA synthesis was performed using the RevertAid H Minus First Strand cDNA Synthesis Kit (Thermo Fisher Scientific, Waltham, MA, United States) according to the manufacturer’s instruction. iTaq Universal SYBR green super mix Bio-Rad was used along with the desired gene-specific oligonucleotide primers (Supplementary Table S3) to evaluate the quantitative expression profile.

*TDH1* (*CAUR_02457*), an ortholog of glyceraldehyde-3-phosphate dehydrogenase (GAPDH) was used as an internal control for normalization of expression. For the constitutive expression profile the ΔCT of *TDH1* was subtracted from the specific gene. The relative expression profiles under drug stresses were calculated using the 2^–ΔΔCT^ method (Livak and Schmittgen, 2001) in comparison to untreated cells. The experiments were performed in biological duplicates and technical triplicates. Statistical significance values were calculated using a two-way ANOVA (uncorrected Fisher’s LSD) in GraphPad Prism 6 software.

### MIC_50_ Determination

Antifungal susceptibility testing was performed according to the broth micro-dilution technique guidelines of the Clinical and Laboratory Standards Institute (CLSI) for AMPB, FLU, voriconazole (VRC), and itraconazole (ITR) (Clinical and Laboratory Standards Institute, 2008). Due to clinical breakpoints not being available for *C. auris*, the interpretation of the breakpoints suggested for yeast was followed. For AMPB, an MIC_50_ of 1 mg/l was considered resistant. *Candida krusei* (ATCC 6258) and *Candida parapsilosis* (ATCC 22019) were used as control organisms (Supplementary Table S1).

## Results and Discussion

### Ploidy Determination and Genome-Wide Sequencing of *C. auri*s

A WGS (close to 200× coverage) of the type strain of *C. auris*, CBS 10913T belonging to the E. Asian geographical clade (Lockhart et al., 2017) was generated using an Illumina MiSeq sequencer (ENA Accession No. PRJEB29190). Final assembly produced a 12.1 Mb genome distributed over 71 contigs with an N50 of 379.6 kb and a GC content of 45%. A total of 5,279 open-reading frames (ORFs) have been identified in the genome of CBS 10913T, which is comparable to published WGSs of other *C. auris* isolates (Muñoz et al., 2018; Rhodes et al., 2018). The genome ploidy of *C. auris* CBS 10913T was confirmed as being haploid using flow cytometry by comparison to a bona fide haploid *S. cerevisiae* strain BY4741 (Supplementary Figure S1). The resulting assembly size of 12.1 Mb and the flow cytometric analysis are in line with previously reported haploid genome sizes of *C. auris* (Lockhart et al., 2017; Muñoz et al., 2018; Rhodes et al., 2018; Bravo Ruiz et al., 2019).

### Identification of Putative ABC Proteins of *C. auri*s

To identify the ABC proteins in *C. auris* CBS 10913T, we used the hidden Markov models (HMMs) profile of ATP-binding domain alignment Pfam (00005) as a query against the 5,279 protein sequences extracted from the *C. auris* CBS 10913T WGS. The initial screening of the HMM profile resulted in 51 sequences as proteins of interest (Supplementary Table S4). After applying cut-off values of 69.4 for the bit scores and 1.3e−19 for the *E*-values, 28 sequences were identified as significant matches and were subjected to further analysis (Table 1 and Supplementary Figure S2). These 28 sequences were confirmed to contain the ABC-specific nucleotide-binding domain (NBD) by ScanProsite^8^ (Table 1).

**TABLE 1:** List of ABC proteins identified in *C. auris* and their molecular size features.

**Protein name**	**Number of amino acids**	**Size in kDa**	**Subfamily**	**Orthologs in *C. albicans***
CAUR_02725	1, 508	169.9196	PDR/ABCG	*CDR1*
CAUR_01285	1, 452	163.474	PDR/ABCG	*SNQ2*
CAUR_01276	1, 486	166.899	PDR/ABCG	*SNQ2*
CAUR_05555	1, 441	163.6461	PDR/ABCG	*CDR4*
CAUR_02773	1, 469	166.9447	PDR/ABCG	*CDR4*
CAUR_04233	1, 264	141.1817	PDR/ABCG	*CDR6(ROA1)*
CAUR_03774	1, 012	113.6363	PDR/ABCG	*ADP1*
CAUR_01852	1, 907	213.6577	MDR/ABCB	*HST6*
CAUR_03761	679	75.3979	MDR/ABCB	*MDL1*
CAUR_04565	1, 432	160.1326	MDR/ABCB	*MDL2*
CAUR_02994	703	78.3624	MDR/ABCB	*ATM1*
CAUR_01188	1, 659	185.6801	MRP/ABCC	*YBT1*
CAUR_00156	1, 469	165.7274	MRP/ABCC	*YCF1*
CAUR_00964	1, 604	180.0822	MRP/ABCC	*MLT1*
CAUR_01719	1, 560	176.1635	MRP/ABCC	*YOR1*
CAUR_00368	1, 424	160.218	MRP/ABCC	*YOR1*
CAUR_03320	1, 566	175.1847	MRP/ABCC	*YCF1*
CAUR_02951	1, 406	158.184	MRP/ABCC	*YOR1*
CAUR_00862	758	85.9342	ALDP/ABCD	*PXA2*
CAUR_04133	810	94.1119	ALDP/ABCD	*PXA1*
CAUR_02351	1, 106	124.1289	ABCF/YEF3	*GCN20*
CAUR_03795	1, 420	157.7549	ABCF/YEF3	*GCN20*
CAUR_04953	751	84.7145	ABCF/YEF3	*NEW1*
CAUR_00824	1, 051	116.4815	ABCF/YEF3	*GCN20*
CAUR_04813	610	68.1726	ABCF/YEF3	*HEF3*
CAUR_04997	617	68.8987	RLI/ABCE	*RLI1*
CAUR_03076	322	36.9176	OTHERS	*CAF16*
CAUR_04824	547	61.7759	OTHERS	*CAF16*

To be classified as an ABC transporter protein, at least one NBD and one TMD must be present in the protein sequence. ABC proteins which lack TMDs are considered as soluble ABC proteins (Prasad et al., 2015). To predict the TMDs among all the putative ABC proteins, we used TOPOCONS and TMPred software, which led to the identification of 20 proteins, which harbor at least one TMD and one NBD; we consider these to be localized to membranes (Supplementary Table S5). The remaining eight proteins lacking a TMD likely are soluble ABC proteins (Supplementary Table S5).

### Phylogenetic Analysis, Domain Organization, and Subfamily Prediction of *C. auris* ABC Proteins

We used the characteristic conserved sequence of NBDs to classify *C. auris* ABC proteins into subfamilies. According to the recent nomenclature suggested by the HUGO gene nomenclature committee^9^, eukaryotic ABC superfamily proteins can be classified into nine subfamilies from ABCA to ABCI (Dean et al., 2001a). However, not all subfamily members are uniformly present in all organisms. For instance, the ABCA subfamily does not exist in yeast, and their presence is limited to plants and some higher organisms (Kovalchuk and Driessen, 2010). ABCI is the most recent subfamily added to this classification, whose existence is confined only to plant taxa (Wasi et al., 2018). Likewise, members of another subfamily, ABCH, are only found in insects and zebrafish (Wasi et al., 2018).

**FIGURE 1:**
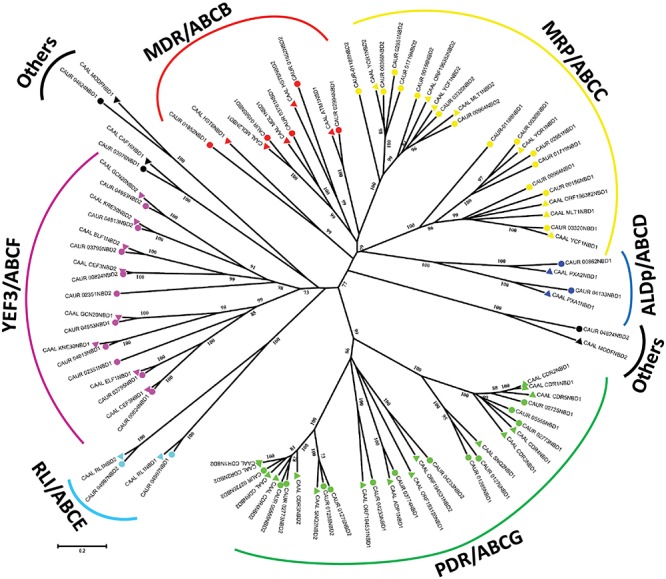
A phylogenetic tree depicting ABC protein subfamilies based on the NBD sequences of *C. auris* (CBS 10913T) and *C. albicans* (SC5314). *C. auris* (circle) and *C. albicans* (triangle) ABC protein NBD sequences were aligned using ClustalΩ. The evolutionary relationship was inferred using the neighbor-joining method, and the evolutionary distances were computed using the Poisson correction method in units of number of amino acid substitutions per site of the MEGA 6.06 package. Reliability of the tree topology was confirmed by bootstrap analysis employing 1,000 replicates. NBD1 represents the N-terminal NBD and NBD2 the C-terminal NBD.

For subfamily predictions, the NBD sequences of putative ABC proteins of *C. auris* were extracted using ScanProsite, and were aligned with the NBD sequences of *C. albicans*. An unrooted phylogenetic tree was constructed using the MEGA6.06 software as described in the section “Materials and Methods.” The well-defined ABC proteins of *C. albicans* (Gaur et al., 2005) allowed us to assign the subfamilies of *C. auris* (Figure 1). The NBD-based phylogenetic analysis revealed that the ABC proteins of *C. auris* clustered into six major subfamilies: (I) pleiotropic drug resistance (PDR)/ABCG, (II) multidrug resistance (MDR)/ABCB, (III) MRP/ABCC, (IV) adrenoleukodystrophy protein (ALDp)/ABCD, (V) RNase-L inhibitor (RLI)/ABCE, and (VI) elongation factor 3 (YEF3)/ABCF. The proteins not segregating with any of these subfamilies were categorized as “Others.” Interestingly, our phylogeny analysis reveals that within the same subfamily N-terminal and C-terminal NBDs of all the proteins form separate clusters. This separation of clusters within a subfamily corroborates previous studies, which suggested that a common ancestor gives rise to each half of the proteins within each subfamily, and also may allocate different functions (Daumke and Knittler, 2001). More recent data also point to this functional asymmetry between N- and C-terminal NBDs (Banerjee et al., 2018). Notably, the presence and the positional order of TMDs and NBDs in each subfamily are specific, and this domain arrangement creates a topological difference among different members of subfamilies. Thus, ABC proteins are clustered into three groups. The first group has a forward topology where the NBD is preceded by the TMD (TMD-NBD); this is observed in the members of the ABCC/MRP, ABCB/MDR, and ABCD/ALDp subfamilies. The second group has a reverse topology where NBD is followed by TMD (NBD–TMD); this is a typical feature of the ABCG/PDR subfamily in fungi and plants. A third group of members of two subfamilies ABCE/RLI and ABCF/YEF3 do not possess any TMD domain and belong to the soluble ABC proteins.

**FIGURE 2:**
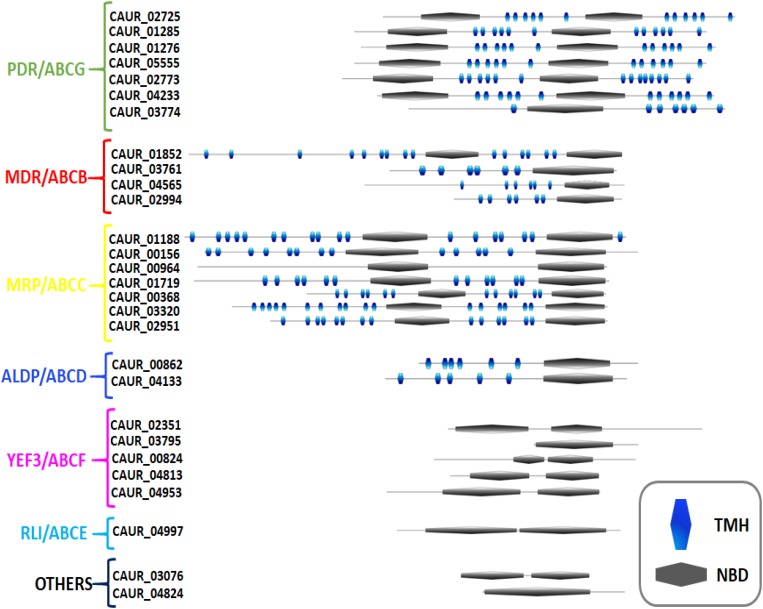
Predicted topology of ABC proteins of *C. auris*. The domain arrangement of *C. auris* ABC proteins was generated using my domain builder tool in PROSITE. The transmembrane helices (TMHs) in transmembrane domain (TMD) were identified with the help of TOPOCONS and nucleotide-binding domain by ScanProsite. The subfamilies were assigned on the guidelines proposed by Human Genome Organization (HUGO).

We utilized the topological distinction to validate our phylogeny-based subfamily clustering of *C. auris* ABC proteins. Using TOPOCONS and TMPRED, the number of transmembrane helices (TMHs) and their arrangement with NBDs were identified (Figure 2 and Supplementary Table S5). Our topology prediction suggests that out of the 20 ABC proteins possessing a TMD, 6 proteins are half-size transporters (containing one NBD and one TMD each), while 14 members can be considered as full transporters because they have 2 TMDs and 2 NBDs each. A further eight proteins do not have a TMD and are thus considered soluble ABC proteins (Figure 2 and Supplementary Table S5).

**FIGURE 3:**
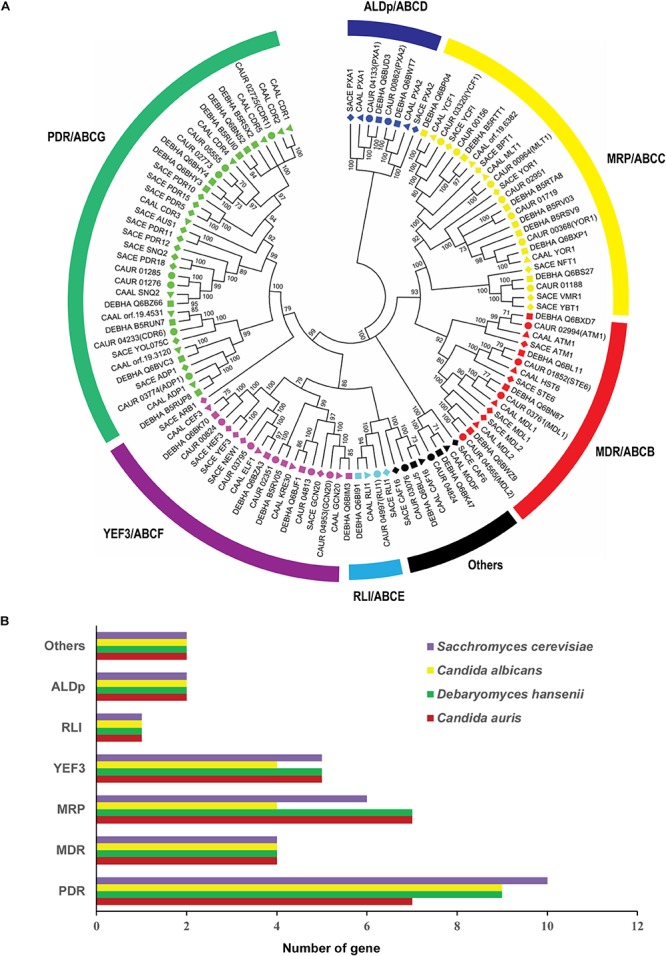
Comparative analysis of *C. auris* ABC proteins with other yeast. **(A)** Comparative phylogenetic relationship of *C. auris* ABC proteins with *C. albicans*, *D. hansenii*, and *S. cerevisiae*. Symbols: circles represent *C. auris* proteins, squares *D. hansenii* proteins, triangles *C. albicans* proteins, and diamonds *S. cerevisiae* proteins. **(B)** The number of ABC proteins in each subfamily in *C. auris*, *D. hansenii*, *C. albicans*, and *S. cerevisiae*. Different subfamilies are plotted on the *y*-axis and the number of genes is indicated on the *x*-axis.

To get better insight into the evolutionary status of *C. auris* ABC proteins, we performed a phylogenetic analysis along with the full ABC proteins of *S. cerevisiae*, *C. albicans*, and *D. hansenii* (Figure 3A). Descriptions of each subfamily of *C. auris* and their comparative analysis are detailed in the following sections.

#### ABCG/PDR Subfamily

ABCG/PDR is ubiquitous in eukaryotes and one of the largest subfamily. Based on combined phylogenetic analysis and topology predictions, we observed that *C. auris* harbors seven ABCG/PDR subfamily members. The number of PDR members varies among different yeasts. While *C. glabrata*, *C. albicans*, and *D. hansenii* have higher numbers, the fission yeast *Schizosaccharomyces pombe* contains only two PDR genes (Lamping et al., 2010; Kumari et al., 2018; Wasi et al., 2018).

Our topology analysis confirmed that all PDR subfamily members of *C. auris* follow the reverse topology (NBD–TMD) arrangement. The full transporters contain two NBD–TMD units following each other (NBD–TMD–NBD–TMD), while half-size transporters possess only one of each (NBD–TMD) (Lamping et al., 2010). Out of the seven PDR members, CAUR_02725, CAUR_02773, CAUR_04233, CAUR_01276, CAUR_01285, and CAUR_05555 are full transporters, while CAUR_03774 is a half-size transporter (Figure 2). Each TMD usually possesses six TMHs; however, in the case of CAUR_02773 protein, its C-terminal TMD is predicted to consist of eight TMHs; this is also observed in the PDR-type ABC member DEBHA_Q6BHY3 of *D. hansenii* (Wasi et al., 2018). Surprisingly, the PDR subfamily in *C. auris* has fewer members compared to other yeast species, such as *S. cerevisiae* (10 members), *C. albicans* (9 members), and *D. hansenii* (9 members) (Figure 3B). Most PDR subfamily members of other yeasts are multidrug transporters. However, only one of the PDR subfamily members (*CDR1*) of *C. auris* has recently been characterized as being directly involved in azole resistance in *C. auris* (Kim et al., 2019; Rybak et al., 2019). The overexpression of PDR transporters correlates well with the emergence of multidrug resistance in *C. albicans*, *C. glabrata*, and other pathogenic fungi (Prasad and Goffeau, 2012). In *S. cerevisiae*, Pdr5 is one of the well-characterized members of this subfamily. It is capable of extruding a wide range of xenobiotics and functions as a promiscuous drug transporter (Jungwirth and Kuchler, 2006; Lamping et al., 2010). *C. albicans* PDR subfamily members *CDR1* and *CDR2* can complement a null mutant of *PDR5* in *S. cerevisiae*, and importantly are also involved in the multidrug resistance phenotype of clinical *C. albicans* isolates (Prasad et al., 1995; Sanglard et al., 1997). Apart from their role in drug transport, members of the PDR subfamily have also shown to impact lipid homeostasis. For instance, Pdr5 in *S. cerevisiae* and Cdr1, Cdr2, Cdr3, and Cdr4 of *C. albicans* are well-characterized lipid translocators (Prasad et al., 2016).

Based on sequence identity with functionally characterized members of other yeasts, the functions of *C. auris* proteins can be predicted. For instance, CAUR_02725 of *C. auris* shows 73 and 68% sequence identity with Cdr1 and Cdr2 of *C. albicans*, respectively, thus forming a subgroup. Notably, our analysis does not reveal an obvious independent homolog of Cdr2 as in *C. albicans* and *C. glabrata*. CAUR_02773 and CAUR_05555 of *C. auris* show 64% and 68% sequence identity, respectively, with Cdr4 of *C. albicans*, and along with DEBHA_Q6BHY3 and DEBHA_Q6BHY4 of *D. hansenii* cluster into a separate subgroup. Interestingly, two proteins, CAUR_01276 and CAUR_01285, of *C. auris* cluster together on a separate branch with Snq2 of *C. albicans*. However, independent blast searches show that both the proteins display 60% sequence identity with Snq2 of *C. albicans*, which strongly suggests that *C. auris* might possess two independent genes coding for Snq2-like proteins, in contrast to *C. albicans* and *D. hansenii* which only possess a single ortholog. An additional copy of *SNQ2* has also been reported in a *C. auris* isolate belonging to the S. African geographical clade, B11221 (Muñoz et al., 2018). A closer examination suggests that these two Snq2-like proteins form a separate branch from that formed by *S. cerevisiae* Snq2 and Pdr18, which perform overlapping functions and are considered paralogs (Godinho et al., 2018). The separate blast searches with these two *S. cerevisiae* proteins show that *C. auris* CAUR_01276 and CAUR_01285 have 55 and 54% sequence identity with Pdr18 and Snq2, respectively. *S. cerevisiae* YOL075C of the PDR subfamily and its homologs *C. albicans* Cdr6 and *D. hansenii* DEBHA_B5RUN7 constitute a distinct cluster, to which CAUR_04233 also belongs; CAUR_04233 has 51% sequence identity with Cdr6. The separation of this cluster of proteins from other PDR subfamily members suggests an early origin, as it is among the few ABCG protein members, that are also observed in primitive eukaryotes like *Batrachochytrium dendrobatidis* and *Encephalitozoon cuniculi* (Kovalchuk and Driessen, 2010). While YOL075C of *S. cerevisiae* remains uncharacterized, its homolog in *C. albican*s Cdr6 has recently been shown to impact drug susceptibility mediated by TOR signaling (Khandelwal et al., 2018).

The only half-size transporter member of the PDR subfamily, CAUR_03774, harbors a TMH_1_–NBD–TMH_6_ topology (Figure 2), and shows a close phylogenetic relationship with Adp1 of *S. cerevisiae* and *C. albicans*. CAUR_03774 has 54% sequence identity with *S. cerevisiae* Adp1, and 67% sequence identity with *C. albicans* Adp1. Similar to Adp1, CAUR_03774 is characterized by the presence of a domain containing epidermal growth factor repeats at its N-terminus, where the first NBD would be situated, if it were a full-size ABC transporter (Figure 2). As with *D. hansenii* and *C. albicans*, there is no homolog of *S. cerevisiae* Aus1 observed in *C. auris* (Wilcox et al., 2002). Our subcellular predictions show that similar to the most closely related PDR subfamily members in *S. cerevisiae*, most of the PDR-type ABC proteins in *C. auris* are probably localized to the plasma membrane (Supplementary Table S6).

#### ABCB/MDR Subfamily

The ABCB/MDR subfamily is characterized by a forward topology, comprising full and half-size transporters. Our analysis revealed that the MDR subfamily of *C. auris* has four ABC transporters: CAUR_01852, CAUR_03761, CAUR_04565, and CAUR_02994 (Figure 1). Notably, CAUR_03761, CAUR_04565, and CAUR_02994 are half-size transporters, while CAUR_01852 is predicted to be a full transporter (TMD–NBD–TMD–NBD) (Figure 2). Unlike the PDR subfamily in *C. auris*, the number of MDR subfamily members is equal to *S. cerevisiae*, *C. albicans*, and *D. hansenii* pointing toward a conserved functional nature across different fungal species (Figure 3). One of the most studied examples of this subfamily is human P-glycoprotein, which is a full transporter, and its overexpression in cancer cells is associated with multidrug resistance (Ambudkar et al., 1992). The only full transporter, CAUR_01852, shows close homology with *S. cerevisiae* Ste6, which transports pheromones, and with *C. albicans* Hst6, which functionally complements Ste6 in *S. cerevisiae* (Raymond et al., 1998). One of the half-size transporters, CAUR_02994, displays high sequence identity 77% and 68% with Atm1 of *C. albicans* and *S. cerevisiae*, respectively. In both species Atm1 is a mitochondrial transporter involved in exporting Fe–S clusters from the inner mitochondrial membrane into the cytosol (Leighton and Schatz, 1995). The other two half-size transporters, CAUR_03761 and CAUR_04565, display an orthologous relationship with Mdl1 and Mdl2, respectively, which are mitochondrial peptide transporters in *S. cerevisiae* (Young et al., 2001; Figure 3A). Our subcellular localization prediction suggests that CAUR_01852 (the Hst6-homolog) is localized to plasma membranes, similar to its homolog in *S. cerevisiae* (Supplementary Table S6). Notably, the other three members of MDR family, the Mdl1-homolog CAUR_03761, the Mdl2-homolog CAUR_04565, and the Atm1-homolog CAUR_02994, might be present on mitochondrial membranes (Supplementary Table S6).

#### ABCC/MRP Subfamily

The ABCC/MRP subfamily is the second largest subfamily of the ABC superfamily found in most eukaryotes and contains only full-length transporters. Our analysis showed that *C. auris* harbors seven putative MRP subfamily members: CAUR_01188, CAUR_00156, CAUR_00368, CAUR_00964, CAUR_01719, CAUR_03320, and CAUR_02951 (Figures 1, 2). Notably, our comparative analysis reveals that the number of MRP subfamily members in *C. auris* (seven members) is equivalent to *D. hansenii* (seven members), but expanded compared to *C. albicans* (four members) and *S. cerevisiae* (six members) (Figure 3B).

Multidrug resistance-associated protein subfamily members form two major clusters: one with CAUR_01188, which aligns with Ybt1, Vmr1, and Nft1 of *S. cerevisiae*, and the second major cluster, which branches into three subgroups (Figures 1, 3A). Out of these three, one subgroup is formed by the CAUR_00368, CAUR_01719, and CAUR_02951 of *C. auris*, and similar to Yor1 of *C. albicans* and *S. cerevisiae*; the second consists of CAUR_00964 and *C. albicans* Mlt1; and the third subgroup contains CAUR_03320 with 67% sequence identity to *C. albicans* Ycf1 and CAUR_00156, which displays similarity to *S. cerevisiae* Ybt1 and *C. albicans* Orf19.6382 (Figure 3A).

This subfamily is also known for the presence of long MRP proteins, which have an extra TMD called the N-terminal extension (NTE) or (TMD)_0_. Usually the (TMD)_0_ consists of five TMHs and hence long MRPs have a (TMD)_0_–(TMD–NBD–TMD–NBD) topology (Figure 2). It is well-documented in *S. cerevisiae* that the (TMD)_0_ of Ycf1 is required for proper localization to the vacuolar membrane (Mason, 2002). Our analysis shows that out of the seven predicted members, two members CAUR_01188 and CAUR_03320 possess a (TMD)_0_–(TMD–NBD–TMD–NBD) topology and are long MRPs (Figure 2). Interestingly, another two MRP members, CAUR_00156 and CAUR_02951, have two additional TMHs at their N-terminus, displaying an unusual TMH_2_–(TMH_6_–NBD–TMH_6_–NBD) arrangement (14 TMHs in total) (Figure 2). However, the phylogenetic analysis clusters CAUR_00156 with *D. hansenii* DEBHA_B5RTT1 and *C. albicans* Orf19.6382, which are predicted to have 17 TMHs, a (TMD)_0_–(TMD–NBD–TMD–NBD) topology (Wasi et al., 2018). CAUR_00368 and CAUR_01719 show a typical (TMD–NBD–TMD–NBD) topology. Notably, no TMDs are predicted for CAUR_00964 by TOPOCONS software, but it appears to have 12 TMDs and a (TMD–NBD–TMD–NBD) according to TMPRED software (Supplementary Table S5).

The members of the MRP subfamily are known to be localized to vacuoles in various yeasts (Snider et al., 2013; Khandelwal et al., 2016) and are well known for their roles in cellular detoxification and sequestration of heavy metal ions in the form of conjugates with glutathione (GSH), glucuronate, or sulfate (Paumi et al., 2007). Recently, our group has shown vacuolar localization for Ybt1 in *S. cerevisiae* and Mlt1 in *C. albicans*, which sequester azoles into the vesicle lumen (Khandelwal et al., 2019). The MRP transporters are also involved in the translocation of bile acids and phosphatidylcholine into the vacuole and affect the regulation of vacuolar fusion (Gulshan and Moye-Rowley, 2011; Sasser and Fratti, 2014; Khandelwal et al., 2016). The other member, yeast cadmium factor (Ycf1) of *S. cerevisiae* helps the cell with detoxification of heavy metal conjugates with GSH, vacuolar fusion, and also affects chronological aging (Paumi et al., 2007; Sasser and Fratti, 2014). Notably, in *C. auris* out of seven MRP subfamily members, three transporters (CAUR_00368, CAUR_01719, and CAUR_02951) lack the extra five TMHs at the very N-terminus (NTE/TMD_0_), and hence are predicted to be localized to the plasma membrane (Supplementary Table S6).

#### ABCD/ALDp Subfamily

Similar to *S. cerevisiae*, *C. albicans*, and *D. hansenii*, the ALDp subfamily of *C. auris* has two members, namely CAUR_00862 and CAUR_04133 (Figures 1–3). Predominantly, members of the ALDp subfamily are half-size transporters with a forward topology (TMD–NBD); in plants also full-length transporters of this subfamily have been described (Wasi et al., 2018). The half-size ALDp transporters turn into functionally complete transporters after hetero- or homo-dimerization (Morita and Imanaka, 2012).

The members of the ALDp subfamily are also known as the peroxisomal ABC transporters, which are involved in fatty acid regulation (Morita and Imanaka, 2012). Our localization predictions suggest that *C. auris* members of this subfamily are also peroxisomal transporters (Supplementary Table S6). Phylogenetic analysis indicates that CAUR_04133 is homologous to Pxa1 (44% identity), while CAUR_00862 is 42% identical with Pxa2 of *S. cerevisiae* (Figure 3A). Pxa1 and Pxa2 are long chain fatty acid transporters in *S. cerevisiae* (van Roermund et al., 2012). The clear evolutionary orthologous relationship between the ALDp subfamily members in various species strongly points toward conserved functions.

#### ABCE/RLI Subfamily

This subfamily belongs to the soluble ABC proteins as its members are devoid of TMD regions and have only two NBDs in tandem (NBD–NBD) (Figure 2). ABCE-subfamily proteins are also known as RLIs because of their ability to inhibit the double-stranded RNA nuclease RNase L. However, these proteins are also present in organisms, which do not have RNase L implying their involvement in other important cellular functions (Wasi et al., 2018). The RLI subfamily has the lowest member count among all ABC proteins (Figure 3B). Indeed, *C. auris* contains a single member, CAUR_04997, belonging to this subfamily. The main distinctive feature of RLI subfamily proteins is the presence of a conserved ferredoxin iron–sulfur cluster (4Fe4S-type) (CX2CX2CX3C) motif (Gaur et al., 2005). RLI-subfamily proteins are implicated in processes such as ribosome biogenesis and translation initiation at the 3′-untranslated region of ORFs (Wasi et al., 2018).

#### ABCF/YEF3 Subfamily

Similar to RLI-type ABC proteins, ABCF/YEF3 subfamily members characteristically have two NBDs (NBD–NBD) and no TMDs (Figure 2), and are also considered soluble ABC proteins. In *C. auris*, this subfamily has five protein members: CAUR_04813, CAUR_02351, CAUR_03795, CAUR_04953, and CAUR_00824. The combined phylogenetic analyses showed that CAUR_00824 forms a tight cluster with *C. albicans* Cef3 and *D. hansenii* DEBHA_Q6BK70, and a wider cluster with its ortholog in *S. cerevisiae*, Yef3 and Hef3 (Figure 3A). It is worth pointing out that except for *S. cerevisiae* with two YEF3-like proteins, most fungi have only a single YEF3 ortholog (Kovalchuk and Driessen, 2010). Yef3 in *S. cerevisiae* is involved in translation elongation by supporting the binding of aminoacyl-tRNA to the ribosome. CAUR_04953 of *C. auris* shows high sequence identity with *S. cerevisiae* Gcn20 (72%) and with *C. albicans* Gcn20 (87%). Gcn20 is one of the most-studied members of the YEF3 subfamily, and it is involved in amino acid biosynthesis through the positive regulation of Gcn2 kinase activity (Yuan et al., 2017). Similar to the other CTG-clade yeasts *C. albicans* and *D. hansenii*, no Arb1-like protein can be detected in *C. auris* (Figure 3A). Arb1 is majorly responsible for maintaining ribosome biogenesis in *S. cerevisiae* (Dong et al., 2005).

#### Others: Unclassified ABC Proteins

This group of ABC proteins comprises members that remain separated from the main subfamilies. It contains soluble ABC proteins, because of the absence of TMDs within their sequences. Our analysis shows that *C. auris* has two proteins in this group, namely CAUR_3076 and CAUR_04824 (Figure 1). Topology predictions show that CAUR_04824 has NBD-NBD architecture, while CAUR_3076 harbors a single NBD (Figure 2). The phylogenetics points to a close relationship between CAUR_04824 and Modf of *C. albicans* with a sequence identity of 50%, while CAUR_3076 seems orthologous to *S. cerevisiae* Caf16, which is part of the CCR4–NOT complex and has a regulatory role in transcription (Liu et al., 2011).

### Expression Analysis Confirms Putative ABC Transporter Genes Transcripts

**FIGURE 4:**
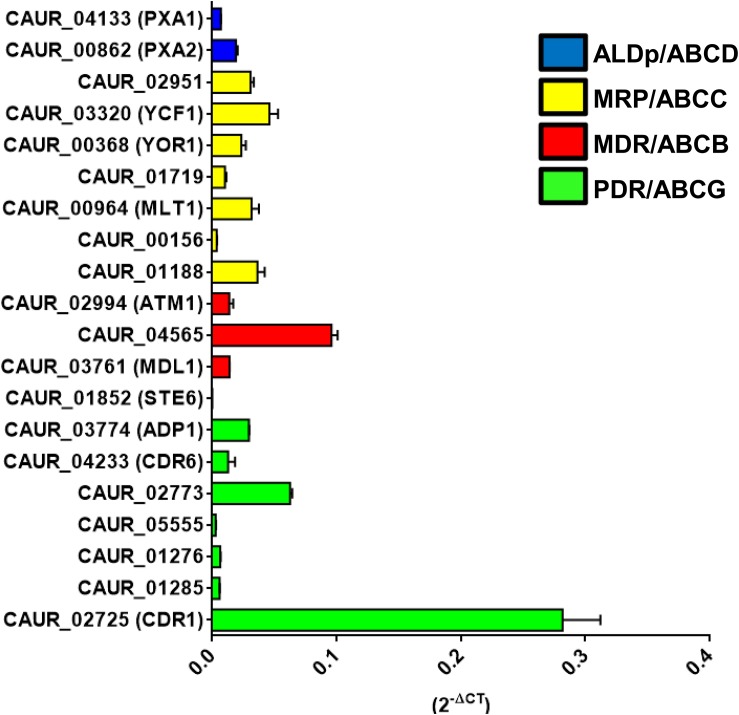
Basal expression levels of ABC transporter genes in *C auris* CBS10913T. The presence of true ABC transporters ORFs was confirmed using qRT-PCR. Expression levels were calculated using the 2^–ΔCT^ methods with the house-keeping *TDH1* gene as internal control. Here 2^–ΔCT^ represents the relative expression of ABC transporter in comparison to *TDH1* gene and its value 1 meaning that the gene expressing equal to the internal control *TDH1*. The values are means ± SE (error bars) from triplicates of biological duplicates (*n* = 6).

As a goal of evaluating the role of ABC proteins as drug transporters in *C. auris* (CBS 10913T), we first assessed the basal expression of all 20 genes encoding membrane-bound ABC proteins. The basal expression profile of all the putative ABC transporter genes was quantified and normalized in comparison to *TDH1* (*CAUR_02457*) gene expression. *TDH1* is a housekeeping gene, which is an ortholog of *GAPDH*, a housekeeping gene commonly used as internal control for qRT-PCR (Kumari et al., 2018). The expression profile of the CBS 10913T strain established the presence of transcripts of all the 20 predicted putative ABC transporter genes, and highlighted a variability of basal expression between them (Figure 4). Although all the ABC transporters showed lower expression than housekeeping gene *GAPDH*, ABC transporters do show constitutive differential expression, which suggests possible different roles. The *C. albicans CDR1*-ortholog *CAUR_02725* showed the highest level of basal expression. *CAUR_01852* (the homolog of *STE6/HST6*) showed the least basal expression among the 20 ABC transporter genes. Notably, similar low expression of *C. glabrata* CAGL0K00363g, an ortholog of *S. cerevisiae STE6* has been reported (Kumari et al., 2018). *SNQ2* orthologs, *CAUR_01276* and *CAUR_01285*, showed similar levels of basal expression. Notably, *CAUR_01852* lacks two nucleotides at positions 3,309 and 3,310 in clade I strains B8441, 6684, and VPCI 479/P/13, and could be considered a pseudogene in these. However, in isolates from all other clades, CBS 10913T, B11220, B11221, and B11243, these residues are present and the ORFs seem to be intact. Considering this and that *CAUR_01852* is transcribed, albeit at low levels, raises the possibility that *CAUR_01852* is functional in *C. auris* isolates from clades II, III, and IV.

### ABC Transporters Display Selective Response to Drug Treatment

**FIGURE 5:**
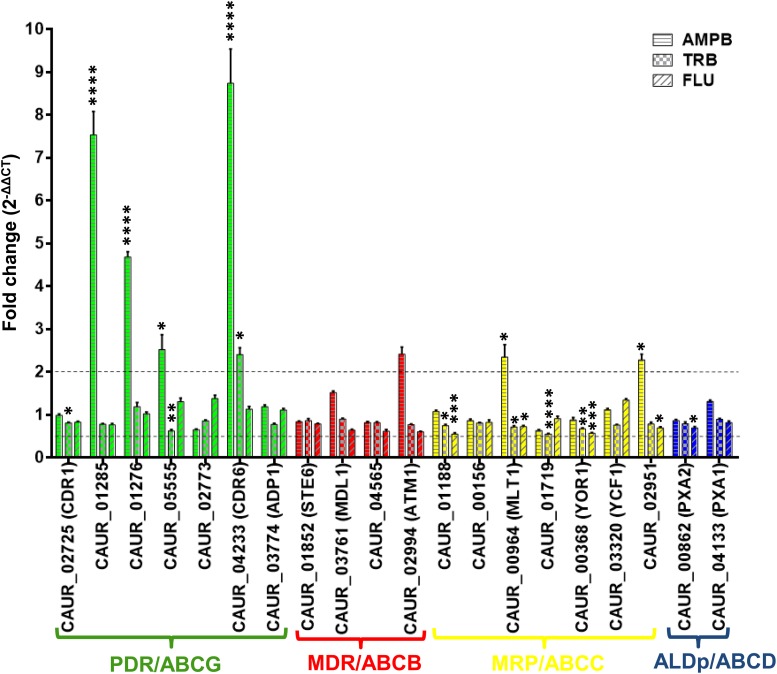
Drug induced expression profiles of various ABC transporter genes in *C. auris*. The expression levels of different genes were checked by qRT-PCR and data were measured by 2^–ΔΔCT^ method in the presence of amphotericin B (0.7 μg/ml), terbinafine (0.75 μg/ml), and fluconazole (6 μg/ml) in comparison to sample with no drug treatment. *TDH1* was used as an internal control. The dotted lines represent the selected biological significant cut off (more than or equal to twofold). 2^–ΔΔCT^ represents the fold change and its values 2, 1, and 0.5 represent twofold up, no change, and twofold down regulation, respectively. Data presented as means ± SE (error bars) from biological replicates with technical triplicates (*n* = 6). *P*-values (^*^p ≤ 0.05, ^∗∗^*p* ≤ 0.001, ^∗∗∗^*p* ≤ 0.0001, and ^****^p ≤ 0.00001) were calculated with two-way ANOVA (uncorrected Fisher’s LSD) using GraphPad prism. *P*-values are for differences from the fold change of 1.

To assess the role of ABC transporters in drug resistance, we examined the changes in ABC transporter transcript levels in response to short-term drug exposure by qRT-PCR. For this, we treated *C. auris* CBS 10913T with different classes of known antifungals, specifically the polyene AMPB, the allylamine TRB, and the triazole FLU. The following MIC_50_ values were used for each drug in YEPD broth for 60 min treatments of the *C. auris* CBS 10913T strain: AMPB at 0.7 μg/ml, TRB at 0.75 μg/ml, and FLU at 6 μg/ml (Supplementary Table S2). The qRT-PCR analysis was performed after the drug treatment and compared with untreated cells (Figure 5). The transcripts of subfamily members were differentially regulated in the presence of the antifungals (Figure 5). PDR subfamily members are well-known drug transporters implicated in clinical drug resistance encountered in different fungi. The observed upregulation of PDR subfamily members of *C. auris* strongly highlights a potential role in drug resistance. Among all the three drugs, AMPB exposure presented maximum response in terms of upregulated expression of selected ABC transporter genes. Particularly, the PDR subfamily genes exhibited higher expression after AMPB exposure, including *CAUR_05555* (one of the homologs of *CDR4*), *CAUR_04233* (homolog of *CDR6*), and both homologs of *SNQ2*: *CAUR_01285* and *CAUR_01276* (Figure 5). An elevated expression of the *SNQ2* homologs in *C. glabrata and S. cerevisiae* has also been observed after AMPB treatment (Rogers et al., 2001); this indicates that *CAUR_01285* and *CAUR_01276* could be involved in drug susceptibility in *C. auris* as well. Incidentally, the more than eightfold higher expression of the *CDR6*-homolog *CAUR_04233* was highest among all ABC transporter transcripts (Figure 5). Interestingly, *CAUR_04233* was also strongly upregulated among transcripts following TRB treatment (Figure 5).

In our present study, ABC genes show maximum transcriptional response to AMPB treatment. This observation also matches the situation in *S. cerevisiae*, in that transcriptional changes in transporters is a major category (10.3% of total genes) affected by AMPB treatment (Zhang, 2002; Agarwal et al., 2003). Also, in *D. hansenii* AMPB treatment resulted in a significant increase in the expression of several ABC transporters (Wasi et al., 2018). This suggests that AMPB treatment-dependent change in expression of ABC transporters is a common response observed in various yeasts.

Considering the well-established role of ABC transporters in membrane lipid homeostasis and the fact that the expression level of ABC transporters can impact AMPB sensitivity, the commonly observed up-regulation of ABC transporters could be a crucial defense mechanism (Smriti et al., 2002; Rockwell et al., 2009; Guan et al., 2010; Nagi et al., 2013; Gulati et al., 2015). The TOR1 pathway also governs AMPB persistence in yeast (Bojsen et al., 2016), indeed, we have recently shown that the ABC transporter Cdr6 can impact TOR signaling and its absence induces the TOR signaling cascade (Khandelwal et al., 2018). This suggests that ABC transporters and TOR signaling are linked and over-expression of ABC transporters could also impact the AMPB sensitivity via TOR signaling.

Strikingly, unlike AMPB and TRB exposure, none of the ABC transporter transcripts showed any significant changes in their expression following FLU treatment (Figure 5). A similar observation was recently reported by Cuomo and co-workers, where they show a lack of response after VRC treatment (Muñoz et al., 2018).

### PDR Subfamily Genes Predominantly Display Elevated Transcript Levels in Drug-Resistant Clinical Isolates of *C. auri*s

The upregulated expression of certain ABC transporters correlates well with an increased resistance to antifungals in clinical isolates of *C. albicans*, *C. glabrata*, and other pathogenic fungi (Sanglard et al., 1999). The upregulation of ABC transporter genes associated with rapid expulsion of antifungal drugs is one of the major strategies adapted by clinical multidrug-resistant strains. To evaluate the relevance of ABC transporter expression levels in multidrug-resistant *C. auris*, we evaluated their expression in three selected clinical isolates of *C. auris* in comparison to *C. auris* (CBS 10913T). These were resistant to AMPB and FLU, and also displayed elevated MIC_50_s to VRC and ITR (Table 2).

**TABLE 2:** *In vitro* susceptibility profile (MIC_50_ values in RPMI media) of *C. auris* strains used in the study to antifungal drugs.

**Strain**	**AMPB**	**FLU**	**VRC**	**ITR**
*C. auris* CBS 10913T	1	1	0.03	0.12
Isolate-1	1	>64	1.00	0.25
Isolate-2	1	>64	0.50	0.13
Isolate-3	4	64	1.00	0.25

*MIC values are given in μg/ml. AMPB, amphotericin B; FLU, fluconazole; VRC, voriconazole; ITR, itraconazole.*

**FIGURE 6:**
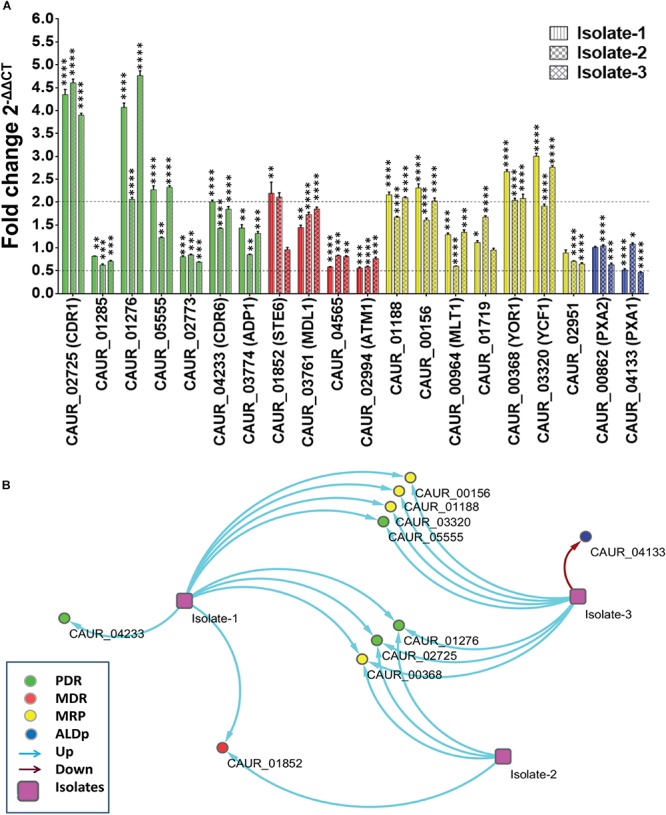
Expression levels of ABC transporter genes in drug-resistant clinical isolates of *C. auris.* **(A)** The expression levels of the various ABC transporter genes in three clinical isolates in comparison to the reference strain CBS 10913T was checked by qRT-PCR. Fold change (2^–ΔΔCT^) measured and presented as means ± SE (error bars) (*n* = 3). *TDH1* was used as an internal control. The dotted lines represent the selected biological significant cut off (more than or equal to twofold). *P*-values (^*^p ≤ 0.05, ^∗∗^*p* ≤ 0.001, ^∗∗∗^*p* ≤ 0.0001, and ^****^p ≤ 0.00001) were calculated with two-way ANOVA (uncorrected Fisher’s LSD) using GraphPad prism. *P*-values are for differences from the fold change of 1. **(B)** Interaction map of ABC transporters in the clinical isolates was made by taking the arbitrary cut off of (more than or equal to twofold) change with the help of Cytoscape software version 3.4.0 (Shannon et al., 2003).

The transcript analysis confirmed the involvement of PDR subfamily members in drug resistance of the emerging pathogenic *C. auris.* Accordingly, the *CDR1*-homolog *CAUR_02725* and the *SNQ2-*homolog *CAUR_01276* displayed higher transcript levels in all tested clinical isolates suggesting a role as a drug exporter in *C. auris* (Figure 6). A role in multidrug resistance including resistance to azoles is well-established for Cdr1 in *C. albicans* and Snq2 in *C. glabrata* (Whaley et al., 2018).

Another PDR subfamily member, *CAUR_04233* (*CDR6* homolog), is (more than or equal to twofold) upregulated in Isolate-1. It also showed a trend toward higher expression in Isolate-2 and Isolate-3, but it was not considered significant since it was below our selected threshold (more than or equal to twofold) of upregulation (Figure 6A). In *C. albicans* the Cdr6 transporter is known as an exporter of xenobiotics (Khandelwal et al., 2018). One of the homologs of *CDR4* (*CAUR_05555*) is upregulated in clinical Isolate-1 and Isolate-3 (Figure 6A), interestingly, elevated expression of *CDR4* in clinical multidrug-resistant *C. albicans* and *C. glabrata* strains has not been reported.

Among the MDR subfamily members, the homolog of *STE6* (*CAUR_001852*) was the only member that exhibited elevated expression in Isolate-1 and Isolate-2 (Figure 6A). Notably, Ste6 is not an established drug transporter, but a dedicated pheromone transporter in *S. cerevisiae* (Kumari et al., 2018). However, one study has reported that Ste6 could also impart resistance to valinomycin in *S. pombe* (Christensen et al., 1997). In *C. glabrata*, the transcripts of a *STE6* homolog (CAGL0K00363g) displayed variable response to drug exposure (Kumari et al., 2018), and also, the human Ste6 homolog, ABCB1, has been shown to extrude xenobiotics in various chemotherapy-resistant tumors (He et al., 2011). Notably, none of the MDR transporters showed more than or equal to twofold downregulation. However, *CAUR_02994* (*ATM1* homolog) of the MDR subfamily showed 1.8- and 1.7-fold downregulation in Isolate-1 and Isolate-2, respectively (Figure 6A). This is in contrast to what was observed after transient exposure of *C. auris* CBS 10913T to AMPB where a more than twofold increase in *CAUR_02994* expression was observed. The homolog of *ATM1* in *C. glabrata* (CAGL0M13739g) displayed raised expression levels following ketoconazole treatment (Kumari et al., 2018).

Recent studies established the roles of MRP subfamily members in fungal drug resistance and virulence (Gulshan and Moye-Rowley, 2011; Khandelwal et al., 2016, 2019). We observed that *CAUR_00368*, which displays close identity with *YOR1*, is upregulated in all three clinical isolates (Figure 6B). In *C. albicans*, Yor1 is involved in expulsion of beauvericin, a natural antimicrobial (Shekhar-Guturja et al., 2016). Upregulation of *YOR1* transcription in *C. auris* indicates a potential role in multidrug resistance of this pathogen. *CAUR_01188/YBT1*, *CAUR_00156/BPT1*, and *CAUR_03320*/*YCF1* show upregulation in two isolates (Isolate-1 and -3). These three transporters are known to be involved extruding various xenobiotics in *S. cerevisiae* (Paumi et al., 2007).

The ALDp subfamily member *CAUR_04133*/*PXA1* transcript is significantly (more than or equal to twofold) down regulated in clinical Isolate-3 (Figure 6B). In clinical Isolate-1, the expression of CAUR_04133 is also decreased (1.93-fold) compared to WT (CBS10913T) (Figure 6A). Coincidently, in *C. glabrata* its homolog *CAGL0M02387* is also downregulated when treated with ketoconazole (Kumari et al., 2018).

## Conclusion

The increasing reports of *C. auris* outbreaks and its emerging resistance to common antifungal pose an alarming clinical situation for hospitals. An established role of ABC transporters in multidrug resistance of different fungal pathogens highlights an essential requirement for understanding the impact these ABC proteins might have in *C. auris.* Here, we have analyzed the full repertoire of ABC proteins of the *C. auris* type strain CBS 10913T.

During the preparation of this manuscript two studies were published (Kim et al., 2019; Rybak et al., 2019) which further highlighted the role of some of the ABC transporters (mainly of PDR family) of *C. auris*. Our study is a comprehensive analysis of total landscape of ABC protein in *C. auris*. Our evaluation identified 28 putative ABC proteins, which is based on phylogeny and domain organization, demonstrated that these could be clustered into the six major ABC transporter subfamilies. Of these, 20 of the putative ABC proteins contain TMDs and are categorized into the four membrane-localized subfamilies. The constitutive expression of these membrane-localized transporters confirmed their presence as true ORFs, while differential expression of the genes encoding ABC transporters implies different biological and clinical relevance. The selective responses of some of these transporters to antifungal drug treatment possibly provide a clue of their relevance in clinically drug resistance mechanisms. The higher expression (more than or equal to twofold) of the *CAUR_02725* (*CDR1*) and *CAUR_01276* (*SNQ2*) ABC transporters in multidrug-resistance clinical isolates of *C. auris* also supports that these transporters may be involved in drug resistance. Our study therefore provides the first complete landscape of ABC transporters of this pathogen, which presents a pivotal platform for further exploration of this important class of proteins and their relevance in multidrug resistance in *C. auris*.

## Data Availability

The datasets analyzed for this study can be found at https://www.ebi.ac.uk/ena/data/view/PRJEB29190 (ENA Accession No. PRJEB29190).

## Author Contributions

RP, NG, MW, and NK conceived and designed the experiments. MW, NK, AJM, RN, and PV performed the bioinformatics analysis. AJM, GR, ZR, AL, and NG carried out the ploidy determination and genome-wide sequencing of *C. auris*. MW and NK carried out the constitutive and drug-induced expression profiling. MW performed the expression analysis of the clinical isolates. RP, NK, MW, RN, and AL analyzed the data and wrote the manuscript. SR and AC provided the clinical isolates and performed the drug profiling experiments. AKM, RP, AML, NG, and AL provided the lab space and reagents for the experiments. All authors read and approved the final manuscript.

## Conflict of Interest Statement

The authors declare that the research was conducted in the absence of any commercial or financial relationships that could be construed as a potential conflict of interest. The reviewer RC declared a past co-authorship with one of the authors RP to the handling editor.

## Footnotes

1 http://hannonlab.cshl.edu/fastx_toolkit/links.html

2 https://www.bioinformatics.babraham.ac.uk/projects/fastqc/

3 http://pfam.sanger.ac.uk/

4 http://hmmer.org/

5 https://blast.ncbi.nlm.nih.gov/Blast.cgi?PAGE=Proteins

6 https://wolfpsort.hgc.jp/

7 http://www.cbs.dtu.dk/services/DeepLoc/

8 http://prosite.expasy.org/scanprosite/

9 https://www.genenames.org/
