# Supplementary material for: ABC Transporter Genes Show Upregulated Expression in Drug-Resistant Clinical Isolates of Candida auris: A Genome-Wide Characterization of ATP-Binding Cassette (ABC) Transporter Genes
Source: Front Microbiol. 2019 Jul 16;10:1445. doi: 10.3389/fmicb.2019.01445 (PMC6647914; doi:10.3389/fmicb.2019.01445)
Supplement: Supplementary file 1 [file Data_Sheet_1.doc]

**ABC Transporter Genes Show Upregulated Expression in Drug Resistant Clinical Isolates of *Candida auris*: a Genome-Wide Characterization of ATP-Binding Cassette (ABC) Transporter Genes**

Mohd Wasi^1,#^, Nitesh Kumar Khandelwal^1,$,#^, Alexander J. Moorhouse2,^,#, Remya Nair^3^, Poonam Vishwakarma^4^, Gustavo BRAVO RUIZ^5^, Zoe K. Ross^2,5^, Alexander Lorenz^5^, Shivaprakash M Rudramurthy^6^, Arunaloke Chakrabarti^6^, Andrew M. Lynn^4^, Alok K. Mondal^1^, Neil A. R. Gow^2,5,7^and Rajendra Prasad^3,*^

^1^School of life Sciences, Jawaharlal Nehru University, New Delhi, India

^$^Current address: Chemistry and Biochemistry, University of Arizona, Tucson, USA

^2^MRC Centre for Medical Mycology, University of Aberdeen, Aberdeen, UK

^Current address: Department of Genetics & Genome Biology, University of Leicester, University Road, Leicester, UK

^3^Amity Institute of Biotechnology and Integrative Sciences and Health, Amity University Haryana, Amity Education Valley, Gurgaon, India

^4^School of Computational and Integrative science, Jawaharlal Nehru University, New Delhi, India

^5^Institute of Medical Sciences, University of Aberdeen, Aberdeen, UK

^6^Department of Medical Microbiology, Postgraduate Institute of Medical Education and Research, Chandigarh, India

^7^School of Biosciences, University of Exeter, Exeter, UK

^#^Authors contributed equally.

*Correspondence: Prof. Rajendra Prasad

rprasad@ggn.amity.edu

**Supplementary Material**

**
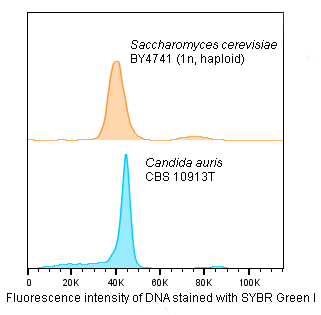
**

**Supplementary Figure S1:** **DNA histogram of flow cytometry analysis showing the ploidy status in *C. auris*:** DNA histograms of flow cytometry analysis as determined by SYBR Green I staining of DNA. *C. auris* CBS 10913T displays the same cell cycle profile as the haploid *Saccharomyces cerevisiae* BY4741 strain. Processing yeast samples for flow cytometry was performed as previously described (Fortuna et al., 2001). Flow cytometry was performed on a BD LSR II flow cytometer (BD Biosciences, San Jose, CA, USA) using an excitation wavelength of 488 nm, SYBR Green I fluorescence was detected with a 530/30 band pass filter. Data were analysed using FlowJo 10.2 software (FlowJo LLC, Ashland, OR, USA).

**Supplementary Figure S2: Identification of ABC domains based on bit scores and the E-values:** Identification of ABC domains among the 5,279 protein sequences extracted from the whole-genome sequence of CBS 10913T based on bit scores and E-values. Red line indicates the E-values, and black line indicates the bit score, dashed line intersection shows the cut-off based on bit score and E-value.

**Supplementary Table S1.** List of strains used in the present study

| **Sl.no** | **Collection no.** | **Species** | **Relevant genotype/Clade** | **Site of isolation** | **Origin/ Reference** |
| --- | --- | --- | --- | --- | --- |
| 1 | CBS10913T  JCM15448^T^ | *Candida auris* | WT E. Asia (Japan) | External ear canal | Satoh K *et al*.(Satoh et al., 2009) |
| 2 | NCCPF470150 | *Candida auris* | Isolate-1 S. Asia (India) | Blood | Present study |
| 3 | NCCPF 470156 | *Candida auris* | Isolate-2 S. Asia (India) | Blood | Present study |
| 4 | NCCPF470114 | *Candida auris* | Isolate-3 S. Asia (India) | Urine | Present study |
| 5 | ATCC 6258 | *Candida krusei* |  | Sputum, Sri Lanka |  |
| 6 | ATCC 22019 | *Candida parapsilosis* |  | Gastro-intestinal tract, Porto Rico | Ashford *et al*.(Ashford, 1928) |
| 7 | BY4741 | *Saccharomyces cerevisiae* | *MAT***a** *his3*∆*1* *leu2*∆*0* *met15*∆*0* *ura3*∆*0* | n/a | Brachmann *et al*.(Brachmann et al., 1998) |

**Supplementary Table S2.** *In vitro* susceptibility profile MIC_50_ values (µg/ml) of *C. auris* CBS10913T in YEPD media used in the study to antifungal drugs.

| **Antifungal** | **MIC_50_ in YEPD (µg/ml)** |
| --- | --- |
| AMPB | 0.70 |
| FLU | 6 |
| TRB | 0.75 |

**Supplementary Table S3.** List of primers used in this study.

FP (forward primer), RP (reverse primer)

| **Primer name** | **Primer sequence (5’- - -3’)** |
| --- | --- |
| CAUR_00156 RT FP  CAUR_00156 RT RP | CATTCTTGGGTCTTCGTGGTGG  CCACAATCACAAGGGTCATCG |
| CAUR_01188 RT FP  CAUR_01188 RT RP | ACACCGGAAACTATTGCCACTG  ACTCTGATGGTCGCCAAAACCG |
| CAUR_00368 RT FP  CAUR_00368RT RP | GACCGCCATTGATGAAGAAAC  GCAAGGGCATTCAATAAGGA |
| CAUR_00824 RT FP  CAUR_00824 RT RP | TGGACAGAGCCAACAGACAG  ACCCAAAAGCCAAGAAACCT |
| CAUR_03761RT FP  CAUR_03761RT RP | TTCCTTGGCACGTACATTGA  GCCGTCTTCAACGACTTCTC |
| CAUR_03774RT FP  CAUR_03774RT RP | TGAATGCTTGGTGAGACTCG  TGGGGGACATCTGTATCCAT |
| CAUR_00862RT FP  CAUR_00862RT RP | AACCAAGGCTCACGAGAGAA  AACATCCAGAACGCCAAAAG |
| CAUR_00964RT FP  CAUR_00964RT RP | TCCGCTTTGAGAAGTCCAGT  CCAACGCTGAGATTGACTGA |
| CAUR_01276RT FP  CAUR_01276RT RP | GCTACGCTAGAAGCGGAGAA  TGGGGATGGTGGGTATCTAA |
| CAUR_01285RT FP  CAUR_01285RT RP | CCTGGTAAGTTGACCGCATT  GCTGAGAACACCAAGGCTTC |
| CAUR_01719RT FP  CAUR_01719RT RP | GCCTGCAAGGCTATCTTGAC  AAAGCACAACGGCAATAACC |
| CAUR_01852RT FP  CAUR_01852RT RP | GTCAACGCTCTGATTGACGA  GCGAAGTGCCAATTATCCAT |
| CAUR_02725RT FP  CAUR_02725RT RP | GGCGTCTTTGTTCAATGGTT  GAGAGGCGATGAAAGCAAAC |
| CAUR_02351RT FP  CAUR_02351RT RP | CGCTACAGTGGGGATTTGTT  TGTGTCCAACACTGGGAAGA |
| CAUR_02773RT FP  CAUR_02773RT RP | CCCTGAAGCTAATCCTGCTG  CTGCGTACGACTTGTGCCTA |
| CAUR_02951RT FP  CAUR_02951RT RP | AATGCAATTGGTTGGGATGT  AAGCAGCGTAAAAGCCGATA |
| CAUR_02994RT FP  CAUR_02994RT RP | GCATTCTTCGACGAGGCTAC  CAAGCCCTGGTGTGTACCTT |
| CAUR_03076RT FP  CAUR_03076RT RP | GGACCTAACGGTGCTGGTAA  CACTCTGTGCCCAAGTACGA |
| CAUR_03320RT FP  CAUR_03320RT RP | GGGAACACCCTTACCTGGAT  GAACGACAGCGAGAGGAAAC |
| CAUR_03795RT FP  CAUR_03795RT RP | ACCGTCACCGAAAACTTGAC  GAATTTTTCCGCATCCTTCA |
| CAUR_04133RT FP  CAUR_04133RT RP | GGAAACTTTCACGGGATTGA  TTGCCTTCTTTTCAGGCACT |
| CAUR_04233RT FP  CAUR_04233RT RP | ATGATATGGCCGTGGCTAAG  GCAGTAACGTCATCGAGCAA |
| CAUR_04562RT FP  CAUR_04562RT RP | GAGGCTCTTGCCAACAAGTC  TCAAAGCAGCACACTTACCG |
| CAUR_04813RT FP  CAUR_04813RT RP | GGAGTTGGAAACCAACCAGA  GGAAACGGAAGGAGAAGACC |
| CAUR_04824RT FP  CAUR_04824RT RP | GTGGATCAACAGCTTGAGCA  CATGCCAGCGTCTAGTTCAA |
| CAUR_04957RT FP  CAUR_04957RT RP | TGTTTGACGAGCCTTCCTCT  ATGTTGATGCCTTCCCTCAC |
| CAUR_04953RT FP  CAUR_04953RT RP | CAGGCCTGAGTTTGAAGAGG  CCAACCACCAGAAAATGCTT |
| CAUR_05555RT FP  CAUR_05555RT RP | ACAACCATCGTCGACATCAA  GCAAATAGACCATGCCGTTT |
| CAUR_02457 RT FP  CAUR_02457 RT FP | AGCTTCTGAGGGCAAATTGA  TTGGCAACGTATTCAACCAA |

**Supplementary Table S4.** HMM profile Output of the predicted 51 ABC protein sequences

| **S.No** | **Protein ID** | **HMM Score** | **E-value** |
| --- | --- | --- | --- |
| 1. | CAUR_01188 | 117.9 | 1.4e-34 |
| 2. | CAUR_00156 | 108.8 | 9.4e-32 |
| 3. | CAUR_01852 | 98.4 | 1.5e-28 |
| 4. | CAUR_03320 | 106.9 | 3.6e-31 |
| 5. | CAUR_00964 | 101.3 | 1.9e-29 |
| 6. | CAUR_03795 | 100.9 | 2.4e-29 |
| 7. | CAUR_04233 | 91.0 | 2.9e-26 |
| 8. | CAUR_04813 | 85.5 | 1.4e-24 |
| 9. | CAUR_00368 | 105.6 | 9e-31 |
| 10. | CAUR_02951 | 97.9 | 2.1e-28 |
| 11. | CAUR_00824 | 82.7 | 1e-23 |
| 12. | CAUR_01719 | 103.9 | 3.1e-30 |
| 13. | CAUR_05555 | 77.3 | 4.7e-22 |
| 14. | CAUR_01285 | 75.8 | 1.4e-21 |
| 15. | CAUR_04953 | 79.2 | 1.2e-22 |
| 16. | CAUR_02725 | 75.3 | 2e-21 |
| 17. | CAUR_01276 | 77.1 | 5.5e-22 |
| 18. | CAUR_02773 | 77.7 | 3.6e-22 |
| 19. | CAUR_02994 | 124.1 | 1.8e-36 |
| 20. | CAUR_03761 | 122.1 | 7.6e-36 |
| 21. | CAUR_02351 | 60.6 | 6.6e-17 |
| 22. | CAUR_04997 | 61.1 | 4.6e-17 |
| 23. | CAUR_03774 | 104.6 | 1.8e-30 |
| 24. | CAUR_04565 | 103.3 | 4.6e-30 |
| 25. | CAUR_04824 | 51.3 | 4.8e-14 |
| 26. | CAUR_00862 | 73.8 | 5.5e-21 |
| 27. | CAUR_04133 | 71.0 | 4.1e-20 |
| 28. | CAUR_03076 | 69.4 | 1.3e-19 |
| 29. | CAUR_00041 | 21.1 | 9.5e-05 |
| 30. | CAUR_02220 | 19.3 | 0.00035 |
| 31. | CAUR_02567 | 13.8 | 0.018 |
| 32. | CAUR_04248 | 12.3 | 0.049 |
| 33. | CAUR_02818 | 13.0 | 0.03 |
| 34. | CAUR_04905 | 18.3 | 0.00073 |
| 35. | CAUR_03379 | 16.6 | 0.0024 |
| 36. | CAUR_04745 | 16.8 | 0.0021 |
| 37. | CAUR_03160 | 9.8 | 0.31 |
| 38. | CAUR_00887 | 15.8 | 0.0042 |
| 39. | CAUR_03600 | 12.8 | 0.036 |
| 40. | CAUR_03725 | 15.8 | 0.0042 |
| 41. | CAUR_01730 | 15.7 | 0.0045 |
| 42. | CAUR_05575 | 12.5 | 0.043 |
| 43. | CAUR_02782 | 15.0 | 0.0072 |
| 44. | CAUR_05227 | 15.0 | 0.0076 |
| 45. | CAUR_01638 | 15.1 | 0.0066 |
| 46. | CAUR_03757 | 15.4 | 0.0056 |
| 47. | CAUR_01893 | 15.8 | 0.0041 |
| 48. | CAUR_01502 | 13.1 | 0.029 |
| 49. | CAUR_03092 | 7.6 | 1.4 |
| 50. | CAUR_03294 | 8.8 | 0.59 |
| 51. | CAUR_02518 | 12.0 | 0.062 |

**Supplementary Table S5.** Analysis of TMDs in ABC proteins of *C. auris*. Numbers of TMHs (transmembrane helices) as predicted by TOPOCONS, TMHMM, or TMPRED.

| **Protein ID** | **TMD Present** | **Numbers of TMHs** | | | **Sub family** |
| --- | --- | --- | --- | --- | --- |
|  |  | **TOPOCONS** | **TMHMM** | **TMPRED** |  |
| CAUR_02725 | Yes | 12 | 12 | 12 | PDR/ABCG |
| CAUR_01285 | Yes | 12 | 11 | 15 | PDR/ABCG |
| CAUR_01276 | Yes | 12 | 11 | 13 | PDR/ABCG |
| CAUR_05555 | Yes | 12 | 12 | 13 | PDR/ABCG |
| CAUR_02773 | Yes | 14 | 11 | 13 | PDR/ABCG |
| CAUR_04233 | Yes | 12 | 13 | 13 | PDR/ABCG |
| CAUR_03774 | Yes | 7 | 8 | 9 | PDR/ABCG |
| CAUR_01852 | Yes | 15 | 12 | 16 | MDR/ABCB |
| CAUR_03761 | Yes | 6 | 4 | 5 | MDR/ABCB |
| CAUR_04565 | Yes | 5 | 4 | 6 | MDR/ABCB |
| CAUR_02994 | Yes | 6 | 5 | 5 | MDR/ABCB |
| CAUR_01188 | Yes | 18 | 18 | 18 | MRP/ABCC |
| CAUR_00156 | Yes | 14 | 11 | 14 | MRP/ABCC |
| CAUR_00368 | Yes | 12 | 10 | 10 | MRP/ABCC |
| CAUR_00964 | Yes | 0 | 12 | 17 | MRP/ABCC |
| CAUR_01719 | Yes | 12 | 10 | 11 | MRP/ABCC |
| CAUR_03320 | Yes | 17 | 14 | 14 | MRP/ABCC |
| CAUR_02951 | Yes | 14 | 11 | 14 | MRP/ABCC |
| CAUR_00862 | Yes | 6 | 3 | 5 | ALDp/ABCD |
| CAUR_04133 | Yes | 6 | 4 | 5 | ALDp/ABCD |
| CAUR_04813 | No | N/A | N/A | N/A | YEF3/ABCF |
| CAUR_02351 | No | N/A | N/A | N/A | YEF3/ABCF |
| CAUR_03795 | No | N/A | N/A | N/A | YEF3/ABCF |
| CAUR_04953 | No | N/A | N/A | N/A | YEF3/ABCF |
| CAUR_00824 | No | N/A | N/A | N/A | YEF3/ABCF |
| CAUR_04997 | No | N/A | N/A | N/A | RLI/ABCE |
| CAUR_03076 | No | N/A | N/A | N/A | Others |
| CAUR_04824 | No | N/A | N/A | N/A | Others |

**Supplementary Table S6. Localization prediction of *C. auris* ABC proteins.**

| **Subfamily** | **Protein ID** | **Wolfpsort** | **DeepLoc** | **Subcellular localization of most closely related ABC protein in *S. cerevisiae*** |
| --- | --- | --- | --- | --- |
| PDR/ABCG | CAUR_02725 | PM | PM | Pdr5 (PM) |
| PDR/ABCG | CAUR_01285 | PM | PM | Snq2 (PM) |
| PDR/ABCG | CAUR_01276 | PM | PM | Snq2 (PM) |
| PDR/ABCG | CAUR_05555 | PM | PM | Pdr15 (PM) |
| PDR/ABCG | CAUR_02773 | PM | PM | Pdr5 (PM) |
| PDR/ABCG | CAUR_04233 | PM | PM | YOL075C (PM) |
| PDR/ABCG | CAUR_03774 | PM | VM | Adp1 (VM) |
| MDR/ABCB | CAUR_01852 | PM | PM | Ste6 (PM) |
| MDR/ABCB | CAUR_03761 | MM | MM | Mdl1 (MM) |
| MDR/ABCB | CAUR_04565 | PM | VM | Mdl2 (MM) |
| MDR/ABCB | CAUR_02994 | MM | MM | Atm1 (MM) |
| MRP/ABCC | CAUR_01188 | PM | VM | Ybt1 (VM) |
| MRP/ABCC | CAUR_00156 | PM | PM | Ycf1 (VM) |
| MRP/ABCC | CAUR_00964 | PM | PM | Bpt1 (VM) |
| MRP/ABCC | CAUR_01719 | PM | PM | Yor1 (PM) |
| MRP/ABCC | CAUR_00368 | PM | PM | Yor1 (PM) |
| MRP/ABCC | CAUR_03320 | PM | PM | Ycf1 (VM) |
| MRP/ABCC | CAUR_02951 | PM | PM | Yor1 (PM) |
| ALDp/ABCD | CAUR_00862 | PM | ER | Pxa2 (Peroxisome) |
| ALDp/ABCD | CAUR_04133 | PM | ER | Pxa1 (Peroxisome) |
| YEF3/ABCF | CAUR_04813 | Cytoplasm | Cytoplasm | Gcn20 |
| YEF3/ABCF | CAUR_02351 | Cytoplasm | Cytoplasm, | Gcn20 |
| YEF3/ABCF | CAUR_03795 | Nucleus | Cytoplasm | New1 |
| YEF3/ABCF | CAUR_04953 | Nucleus | Cytoplasm | Gcn20 |
| YEF3/ABCF | CAUR_00824 | Cytoplasm | Cytoplasm | Yef3/Hef3 |
| RLI/ABCE | CAUR_04997 | Cytoplasm | Nucleus | Rli1 (Nucleus) |
| Others | CAUR_03076 | Nucleus | Cytoplasm | Caf16 |
| Others | CAUR_04824 | MM | MM | Caf6 |

To predict the subcellular localization of ABC transporters we used WoLF PSORT (https://wolfpsort.hgc.jp/), and DEEPLOC (<http://www.cbs.dtu.dk/cgi-bin/webface2.fcgi?jobid=5B82D9FF000038BB3A26039D& wait=20>) software.

PM (Plasma membrane), VM (Vacuole membrane), MM (Mitochondrial membrane), ER (Endoplasmic reticulum)

**References**:

Ashford, B. K. (1928). Certain Conditions of the Gastro-Intestinal Tract in Porto Rico and Their Relation to Tropical Sprue. *Am. J. Trop. Med. Hyg.* s1-8, 507–538. doi:10.4269/ajtmh.1928.s1-8.507.

Brachmann, C. B., Davies, A., Cost, G. J., Caputo, E., Li, J., Hieter, P., et al. (1998). Designer deletion strains derived from Saccharomyces cerevisiae S288C: a useful set of strains and plasmids for PCR-mediated gene disruption and other applications. *Yeast* 14, 115–132. doi:10.1002/(SICI)1097-0061(19980130)14:2<115::AID-YEA204>3.0.CO;2-2.

Fortuna, M., Sousa, M. J., Côrte-Real, M., Leão, C., Salvador, A., and Sansonetty, F. (2001). Cell cycle analysis of yeasts. *Curr. Protoc. Cytom.* 13, 11.13.1-11.13.9. doi:10.1002/0471142956.cy1113s13.

Satoh, K., Makimura, K., Hasumi, Y., Nishiyama, Y., Uchida, K., and Yamaguchi, H. (2009). Candida auris sp. nov., a novel ascomycetous yeast isolated from the external ear canal of an inpatient in a Japanese hospital. *Microbiol. Immunol.* 53, 41–44. doi:10.1111/j.1348-0421.2008.00083.x.
